# Implementation of an extracorporeal resuscitation (ECPR) program for out-of-hospital cardiac arrest in Stockholm, Sweden: Feasibility, safety, and outcome

**DOI:** 10.1016/j.resplu.2024.100596

**Published:** 2024-03-07

**Authors:** Lis Frykler Abazi, Andreas Liliequist, Felix Böhm, Magnus Hedberg, Moa Simonsson, Anders Bäckman, Malin Ax, Frieder Braunschweig, Linda Mellbin, Rickard Linder, Leif Svensson, Juliane Jurga, Per Nordberg, Mattias Ringh, Sune Forsberg, Jacob Hollenberg

**Affiliations:** a Department of Clinical Science and Education, Södersjukhuset, Center for Resuscitation Science, Karolinska Institutet, Sweden; b Function Perioperative Medicine and Intensive Care, Karolinska University Hospital, Stockholm, Sweden; c Department of Clinical Sciences, Danderyd Hospital, Karolinska Institutet, Stockholm, Sweden; d Capio Rapid Response Cars and Perioperative Medicine & Intensive Care, Karolinska University Hospital and Department of Physiology and Pharmacology, Karolinska Institutet, Sweden; e Department of Medicine, Solna, Karolinska Institutet, Stockholm, Sweden; f Department of Cardiology, Heart and Vascular Center, Karolinska University Hospital, Stockholm, Sweden

**Keywords:** Cardiac arrest, Refractory, OHCA, ECPR

## Abstract

**Background:**

The aim of this study was to evaluate the implementation of a novel extra corporeal cardiopulmonary (ECPR) program in the greater Stockholm area with focus on feasibility, safety aspects and clinical outcomes.

**Methods:**

Prospective observational study of ECPR program including patients with OHCA from January 2020 to December 2022, fulfilling ECPR criteria: age 18–65 years, initial shockable rhythm or pulseless electrical activity, witnessed arrest, bystander cardiopulmonary resuscitation and refractory arrest after three cycles of advance cardiac life support. The predefined time threshold from collapse to extracorporeal membrane oxygenation (ECMO) initiation was set at 60 min.

**Results:**

We included 95 patients. Of these, 22/95 (23%) had return of spontaneous circulation before ECMO initiation, 39/95 (41%) were excluded for ECMO and 34/95 (36%) had ECMO initiated out of which 23 patients were admitted alive to the ICU. ECMO-initiation within 60 min was met in 9%. In 6 patients vascular access was complicated, 2 patients had severe bleeding at access site requiring intervention. Survival to discharge among all cases was 25% (24/95). Among patients admitted to ICU on ECMO 39% (9/23) survived to discharge, of these 78% had cerebral performance category scale score 1–2 within 12 months. 8 out of 9 survivors had time from OHCA to ECMO-initiation >60 min.

**Conclusion:**

The implementation of an ECPR protocol was feasible without any major, unexpected safety aspects but did not meet the intended target time intervals. Despite this, survival rates were similar to previous studies although most survivors had >60 min to ECMO-initiation.

## Introduction

Out-of-hospital cardiac arrest (OHCA) is a major cause of death with more than 300.000 victims annually in Europe and America respectively.^1^, ^2^ Approximately only 25% of the OHCA patients, where resuscitation efforts have been initiated by the emergency medical services (EMS), achieve return of spontaneous circulation (ROSC) and are admitted to hospital alive.^3^ There is an increasing interest among a group of younger OHCA victims who do not achieve ROSC despite having characteristics usually associated with high survival rates. Studies indicate that this group of refractory OHCA patients often have a high burden of coronary artery disease.^4^, ^5^ In this context, extracorporeal cardiopulmonary resuscitation (ECPR), which means that extracorporeal membrane oxygenation (ECMO) is initiated during ongoing cardiopulmonary resuscitation (CPR), has emerged as a potential bridge to therapy of the precipitating cause.

Current guidelines on the use of ECPR in selected OHCA patients are based on low-quality evidence.^6^, ^7^ Three recently published randomised controlled trials present conflicting results regarding the potential survival benefits of this ECPR treatment strategy.^8^, ^9^, ^10^ While Yannopoulos et al.^8^ found a considerable improvement in survival in a selected group of patients with shockable rhythms, the improvement found by Belohlavek et al.^9^ in a more heterogenous OHCA population was smaller and non-significant. The third and most recent study by Suverein et al.^10^ found no statistically significant difference in an ECPR strategy in refractory ventricular fibrillation. Therefore, how and when ECPR should be utilized is still debated and study results vary depending on patient selection as well as the capacity of the health care system where it is implemented.^11^, ^12^ The results and experiences on ECPR are difficult to compare and generalize and therefore important to study in various medical systems with specific system factors considered.

The aim of this prospective observational study was to evaluate the implementation of an ECPR program for refractory OHCA in the greater Stockholm area with focus on feasibility, safety aspects and clinical outcomes.

## Methods

### Setting

This observational study reports findings from the implementation of an ECPR protocol in the greater Stockholm area, Sweden with a population of 2.4 million^13^ and an area of 6 514 km^2^.^14^ The EMS share one common dispatch centre and one common two-tier EMS where the first tier can provide ACLS and the second tier consists of three physician-staffed rapid response cars and two helicopters, capable of providing more advanced ACLS. Access to physician-staffed units is reduced at night as only one of the three physician-staffed unites operate at 21:00 to 07:00. ECMO is offered at one of six hospitals, Karolinska University Hospital, and all emergency healthcare is publicly financed. A more in-depth explanation of the organisational prehospital and in-hospital setting for this study can be found in the supplemental digital content.

### Patients

Pre-hospital criteria to activate the ECPR-team (eTable 1) were: 1) witnessed OHCA, 2) CPR initiated within 5 min, 3) ventricular fibrillation (VF)/pulseless ventricular tachycardia (pVT) or pulseless electrical activity (PEA) as initial rhythm, 4) age 18–65 years, 5) continued cardiac arrest following 6 min of ACLS and 6) no known significant malignancy or severe neurological disease. If all these criteria were present the patient was an eligible ECPR-candidate and the ECPR alert was triggered.

At hospital arrival a blood gas was retrieved and a lactate level of 15 mmol/L or higher was chosen as a relative cut off for exclusion together with a set of relative exclusion criteria, time from collapse to ECMO-initiation >60 min being the most important one (eTable 1).

This study therefore includes patients that achieved ROSC before ECMO, patients without ROSC and where ECMO was declined or not initiated, as well as patients where ECMO was initiated. The ECPR program was initiated in January 2020 and the current study includes consecutive patients until December 2022.

### Treatment prior to ECPR protocol implementation and establishment of protocol

Prior to the start of the project, ECPR was rarely initiated for OHCA, and the primary treatment strategy was prehospital treatment on site. Transportation to the emergency department was done at the discretion of the attending staff. The ECPR protocol changed this by defining the eligible patients and enabling early direct transportation to the coronary catheterization laboratory at a dedicated ECPR-team. With the implementation of the ECPR protocol the physician staffed unit as well as the helicopters were equipped with the same mechanical compression device (LUCAS3^TM^, Jolife AB/Stryker. Lund, Sweden). Details on prior treatment and establishment of ECPR chain including a pre-study, inclusion of stakeholders and education as well as protocol are further described in the supplemental digital content.

### Outcomes

Feasibility outcomes included the proportion of correctly identified patients, the proportion of patients arriving at the hospital within different time frames, time frames spent at scene, time to hospital arrival, time from collapse to cannulations and ECMO-initiation and the proportion of patients with successful cannulation. Feasibility also included reporting of severe logistic challenges and problems both pre- and in-hospital.

Safety outcomes included serious adverse events defined as all reported events that lead to potential life-threatening status or death.

Clinical outcomes included timing and duration of key interventions, the proportion of patients who underwent angiography, PCI, target temperature management, as well as survival and neurological function measured as the best cerebral performance category (CPC) score during the first year. (eTable 2).

### Data collection

Data collection in the present study derives from a study-specific prehospital and a hospital case report form (CRF), a pre-hospital EMS database and electronic medical records. The prehospital CRF was completed by the second-tier unit at the time of the cardiac arrest and included prehospital arrest characteristics and timing of events. Upon arrival at the coronary catheterization lab a second study specific hospital CRF was completed by a member of the ECPR-team including data on timing of interventions and events related to the coronary catheterization lab. Both CRFs collected data on protocol deviations and adverse events. Data on comorbidities, aetiology, hospital durations and interventions and outcomes were collected using electronic medical records. Data on EMS arrival time was retrieved from the prehospital electronic medical records. In cases where data was missing in the CRFs the electronic medical records were screened for these details. Patients with missing data points were only excluded for the relevant analyses. A more detailed description of the data collection including the CRFs used can be found in the supplemental digital content.

### Statistical analysis

Categorical variables are presented as counts and proportions and continuous variables as medians and quartiles. Considering the descriptive nature of the study and the lack of control group, no statistical analysis was performed. All calculations were performed using IBM SPSS version 28.

### Ethics

Ethical approval was obtained by the Swedish Ethical Review Authority, DNR: 2017/285–31,

## Results

### Patients

**Fig. 1:**
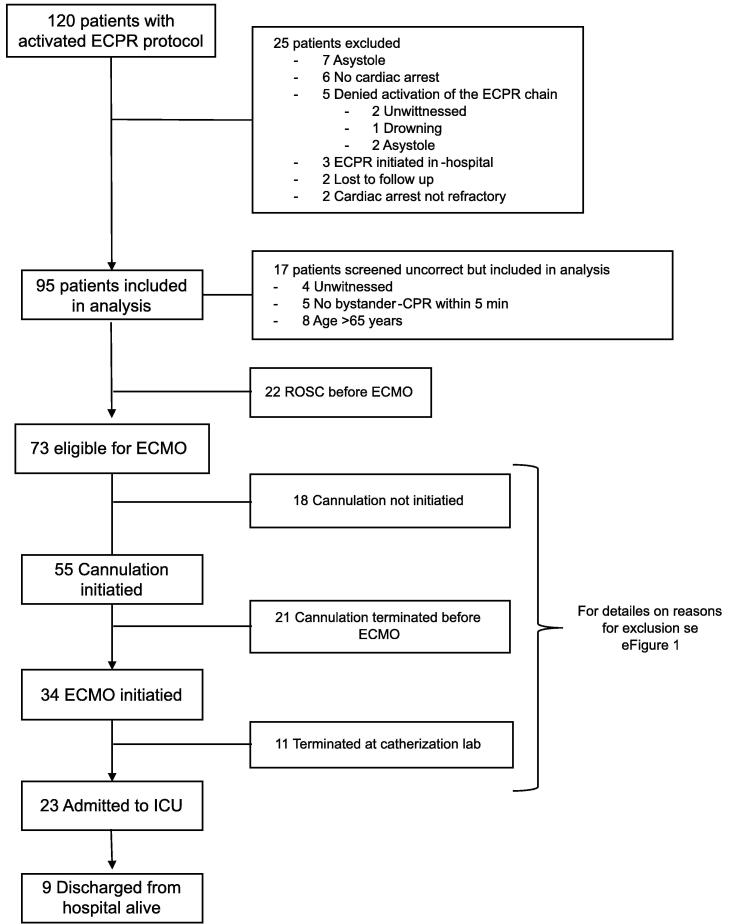
Flowchart of patient inclusion.

Between January 2020 to December 2022 the ECPR protocol was activated for a total of 120 eligible patients. Among these, 25 were excluded for not meeting ECPR-criteria (Fig. 1). A total of 95 individuals were included in the present study. Inclusion rate varied over time (eFigure 1) with a sharp decrease during the first year of the COVID-19 pandemic, which broke out just two months after the launch of the new protocol.

### Baseline characteristics and critical time intervals

The detailed characteristics and past medical history of the population are found in Table 1. Median age was 55 years and 15 patients (16%) were female. Two-thirds of the population, 65 patients, had VF/pVT as the initial rhythm, the remaining had PEA as the first rhythm. Hypertension, smoking and obesity were more prevalent among patients with an initial rhythm of PEA while a history of heart disease was more prevalent among patients with VF/pVT. Patients with an initial rhythm of VF/pVT more often had cardiac cause to the arrest (*N* = 64, 98%) compared to PEA where cardiac cause (*N* = 7, 23%), aortic dissection (*N* = 7, 23%) and pulmonary embolism (*N* = 8, 27%) were about as common. One patient’s cardiac arrest occurred in association with severe COVID-19 while another patient tested positive for SARS-COV2 but without clinical symptoms of COVID-19.

**Table 1:** Baseline characteristics.

	Total population *N* = 95	VT/VF *N* = 65	PEA *N* = 30
Age (median [IQR])	55 [49–62]	54 [48–62]	58 [49–63]
Gender female (%)	15 (16%)	7 (11%)	8 (27%)
Prehospital characteristics			
Witnessed, N (%)	91 (96%)	62 (95%)	29 (97%)
Bystander CPR, N (%)	89 (94%)	61 (94%)	28 (93%)
VF/VT, N (%)	65 (68%)	65 (100%)	0 (0%)
Location at home, N (%)	46 (48%)	30 (46%)	16 (59%)
Total number of shocks (median [IQR])	4 [1–7]	6 [4–8]	0 [0–1]
Total adrenaline dose (mg) (median [IQR])	5 [3–6]	5 [3–6]	5 [4–6]
Amiodarone total dose (mg) (median [IQR])	220 [0–450]	308 [0–450]	0 [0–0]
Medical history, N (%)			
Heart disease	19 (22%)	16 (25%)	4 (14%)
Stroke	2 (2%)	1 (2%)	1 (4%)
Hypertension	32 (35%)	18 (29%)	14 (50%)
Hyperlipidaemia	14 (15%)	10 (16%)	4 (14%)
Diabetes	10 (11%)	7 (11%)	3 (11%)
Renal disease	4 (4%)	3 (5%)	1 (4%)
Respiratory disease	5 (6%)	4 (6%)	1 (4%)
Cancer	4 (4%)	3 (5%)	1 (4%)
Smoking	18 (23%)	11 (20%)	7 (33%)
Obesity	19 (31%)	13 (28%)	6 (40%)
Alcoholism	5 (6%)	4 (7%)	1 (4%)
Cause of cardiac arrest, N (%)			
cardiac	71 (75%)	64 (98%)	7 (23%)
pulmonary embolism	8 (8%)	0 (0%)	8 (27%)
aortic dissection	7 (7%)	0 (0%)	7 (23%)
cerebrovascular insult	2 (2%)	1 (2%)	1 (3%)
other	3 (3%)	0 (0%)	3 (10%)
unknown	4 (6%)	0 (0%)	4 (13%)

Time intervals can be found in Table 2. Median time from cardiac arrest to hospital arrival was 45 min [IQR 36–54 min] and 45 patients (49%) arrived at hospital within 45 min from the cardiac arrest. Median time from hospital arrival to ECMO-initiation was 33 min [IQR 27 – 44 min]. ECMO-initiation occurred in median 77 min [IQR 66 – 90 min] from cardiac arrest. Three patients (9%) had ECMO initiated within 60 min from cardiac arrest.

**Table 2:** Outcomes, eligibility, and time intervals.

	Total population *N* = 95	VT/VF *N* = 65	PEA *N* = 30
Time from: (min, median [IQR])			
cardiac arrest to EMS arrival	8 [6 –13]	10 [5–13]	9 [5–11]
cardiac arrest to arrival of secondary unit	15 [9–20]	15 [10–20]	13 [8–20]
EMS arrival to transportation	24 [18–29]	24 [18–27]	24 [18–30]
departure to hospital arrival	11 [8–16]	10 [8–15]	15 [8–20]
cardiac arrest to hospital arrival	45 [36–54]	45 [38–55]	42 [32–52]
hospital arrival to ECMO start	33 [27–44]	33 [26–42]	39 [30–47]
cardiac arrest to ECMO start	77 [66–90]	78 [67–90]	73 [66–95]
cardiac arrest to ROSC for those with ROSC	32 [17–44]	31 [17–39]	88 [N = 2]
Prehospital, N (%)			
Correctly identified patients	78 (82%)	54 (83%)	24 (80%)
Time from EMS arrival to transportation within:			
<10 min	2 (3%)	1 (2%)	1 (6%)
10–20 min	19 (29%)	14 (30%)	5 (28%)
20–30 min	28 (43%)	21 (45%)	7 (39%)
30–40 min	15 (23%)	10 (21%)	5 (28%)
>40 min	1 (2%)	1 (2%)	0 (0%)
Time to hospital arrival within:			
30 min from cardiac arrest	9 (10%)	5 (8%)	4 (14%)
30–45 min from cardiac arrest	36 (39%)	25 (40%)	11 (39%)
45–60 min from cardiac arrest	42 (46%)	30 (47%)	12 (43%)
60–75 min from cardiac arrest	5 (5%)	4 (6%)	1 (4%)
In-hospital, N (%)	*N* = 33	*N* = 24	*N* = 9
Time from hospital arrival to ECMO start within:^a^			
<15 min	1 (3%)	0 (0%)	1 (11%)
15–30 min	12 (36%)	11 (46%)	1 (11%)
30–45 min	14 (42%)	9 (38%)	5 (56%)
>45 min	6 (18%)	4 (17%)	2 (22%)
Time from cardiac arrest to ECMO start within:^a^			
<60 min from cardiac arrest	3 (9%)	3 (13%)	0 (0%)
60–75 min from cardiac arrest	9 (27%)	4 (17%)	5 (56%)
75–90 min from cardiac arrest	12 (36%)	11 (46%)	1 (11%)
>90 min from cardiac arrest	9 (27%)	6 (25%)	3 (33%)
No ROSC before hospital arrival, N (%)	*N* = 73	*N* = 45	*N* = 28
Lactate level <15^b^	39 (57%)	30 (73%)	9 (33%)
Successful ECMO-initiation	34 (47%)	24 (53%)	10 (36%)

a *One patient missing*.

b Five patients missing.

### Feasibility and safety aspects

**Fig. 2:**
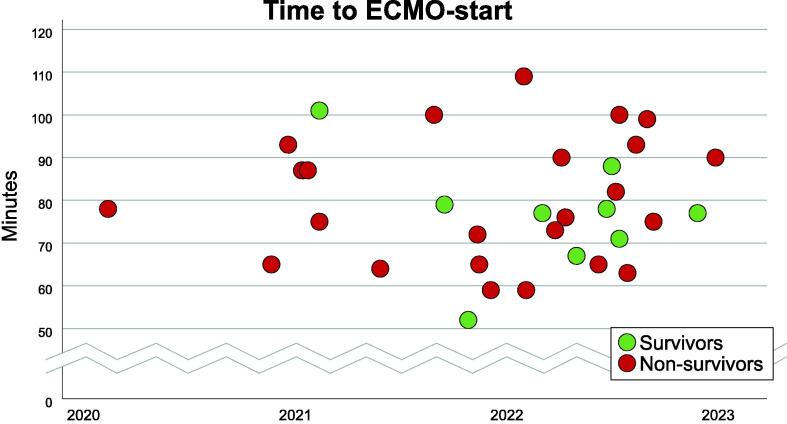
Time to ECMO for survivors and non-survivors over time.

Regarding feasibility aspects the proportion of correctly identified patients according to prespecified ECPR-criteria was 82% (*N* = 78) of the 95 patients included in the analysis. The maximum stipulated time frame of <60 min from cardiac arrest to ECMO-initiation was only met in three (9%) of the 34 patients where ECMO was initiated (Fig. 2).

Few major adverse events were reported during the study period and a list of adverse events is presented in Table 4 with a detailed description in eTable 3. The most common prehospital adverse event was potential damage caused by CPR: haemorrhage in the pleural space, liver laceration and pulmonary bleeding (*N* = 5/95, 5%). During cannulation and ECMO-initiation, the most reported adverse events were difficult access (*N* = 6/52, 12%) followed by severe bleeding at access site requiring intervention (*N* = 2/53, 4%) and insufficient ECMO-flow at ECMO initiation (*N* = 2/52, 4%). There was one case (*N* = 1/52, 2%) of air embolism. The most common reported adverse events at the ICU were renal failure requiring dialysis (*N* = 6/23, 26%) and severe bleeding requiring transfusion (*N* = 4/23, 17%).

### Outcomes, in-hospital interventions

In total, 22/95 patients (23%) achieved ROSC prior to or at the time of arrival at the coronary catheterisation lab without initiation of ECMO, and the median time to ROSC was 32 min [IQR 17–44 min]. Among these, 15/22 patients (68%) survived to hospital discharge. For patients with ROSC, survival to discharge was higher for VF/pVT (*N* = 15/20, 75%) compared with PEA (*N* = 0/2, 0%).

In total 73 patients (77%) did not achieve ROSC before arrival at the coronary catheterization lab. In 39 (53%) of these, cannulation or ECMO following cannulation, was not initiated (Fig. 1). The major reasons were lactate >15 (*N* = 26, 67%) and time from collapse to ECMO-initiation >60 min (*N* = 29, 74%). Reasons for no cannulation or no ECMO are presented in detail in eFigure 2. In many cases the final decision was an overall assessment at the discretion of the attending physicians.

Of the 73 patients who did not achieve ROSC before ECMO, 34 patients (47%) were cannulated and ECMO was initiated. In 11 cases, ECMO was terminated at the coronary catheterization lab before admittance to ICU. The most common reasons were non-working ECMO-circuit (*N* = 2, 18%) and aortic dissection discovered after ECMO-initiation (*N* = 2, 18%).

A total of 23 patients were admitted alive to the ICU on ECMO and 9 patients of these (39%) were discharged alive, of which 7 (30%) had a best positive neurological status of CPC 1–2 within 12 months after the arrest. Among patients where ECMO was initiated survival to discharge was 37% in patients with VF and 50% among PEA. Overall survival for VF patients was 34% (*N* = 22/65) and for PEA 7% (2/30). Only 1 of 9 survivors had ECMO-initiation below the stipulated maximum time frame of <60 min from cardiac arrest (Fig. 2).

Most patients underwent coronary angiography (*N* = 27/34, 79%) but only patients with VF/pVT had findings motivating treatment with PCI; thus PCI was only performed among patients with VF/pVT. The left anterior descending coronary artery was engaged in more than half of the PCI cases (*N* = 7/12, 58%) (Table 3). Duration of ECMO, days in ICU and hospital were longer for survivors compared to non-survivors (Table 3).

**Table 3:** Interventions and treatment details for ECMO-initiated, and survival.

	Total population *N* = 34	VT/VF *N* = 24	PEA *N* = 10
Lactate on arrival, median [IQR]	13 [11–15]	13 [11–14]	15 [12–17]
Underwent coronary angiography, N (%)	27 (79%)	20 (83%)	7 (70%)
PCI, N (%)	12 (35%)	12 (50%)	0 (0%)
Left main / LMCA	3 (25%)	3 (25%)	0 (0%)
Left anterior descending / LAD	7 (58%)	7 (58%)	0 (0%)
Left circumflex / LCX	4 (33%)	4 (33%)	0 (0%)
Right coronary artery / RCA	3 (25%)	3 (25%)	0 (0%)
ECMO terminated before ICU, N (%)	11 (32%)	5 (21%)	6 (60%)
Admitted alive to ICU, N (%)	23 (68%)	19 (79%)	4 (40%)
ECMO duration days, median [IQR]			
Survivors	3 [3–8]	3 [3–4]	9 (*N* = 2)
Deceased	1 [1–3]	1 [1–3]	1 (*N* = 2)
ICU length of stay, days median [IQR]			
Survivors	11 [5–31]	9 [5–17]	36 (*N* = 1)
Deceased	1 [1–2]	1 [1–3]	1 (*N* = 2)
Hospital length of stay, days median [IQR]			
Survivors	28 [18–38]	24 [16–36]	45 (*N* = 1)
Deceased	1 [1–2]	1 [1–3]	1 (*N* = 2)
Survival and neurological function			
ROSC before ECMO, N (%)	*N* = 22	*N* = 20	*N* = 2
Survival ICU discharge^a^	16 (76%)	16 (84%)	0 (0%)
Survival hospital discharge	15 (68%)	15 (75%)	0 (0%)
Survival 6 months	14 (64%)	14 (70%)	0 (0%)
Best CPC within 12 months			
1/1	13 (59%)	13 (65%)	0 (0%)
2/2	0 (%)	0 (%)	0 (0%)
3/3	1 (5%)	1 (5%)	0 (0%)
4/4	0 (%)	0 (%)	0 (0%)
ECMO-initiated and admitted to ICU, N (%)	*N* = 23	*N* = 19	*N* = 4
Survival ICU discharge	9 (39%)	7 (37%)	2 (50%)
Survival hospital discharge	9 (39%)	7 (37%)	2 (50%)
Survival 6 months^b^	8 (35%)	6 (33%)	2 (50%)
Best CPC within 12 months			
CPC 1	7 (30%)	5 (26%)	2 (50%)
CPC 2	0 (0%)	0 (0%)	0 (0%)
CPC 3	2 (9%)	2 (11%)	0 (0%)
CPC 4	0 (0%)	0 (0%)	0 (0%)

a One patient that survived to discharge was conscious and stable at admittance to hospital and did not require ICU care.

b One patient lost to follow up because of transfer to other country. This patient however had CPC 1 at the time of transfer 2 months after cardiac arrest.

**Table 4:** Reported adverse events for patients treated with ECMO.

	Total population	VT/VF	PEA
Prehospital (all ECPR alerts)	***N* = 95**	***N* = 65**	***N* = 30**
Failure of intubation	0 (0%)	0 (0%)	0 (0%)
Failure to apply mechanical compression	1 (1%)	1 (2%)	0 (0%)
Complications potentially caused by CPR*	5 (5%)	5 (8%)	0 (0%)
Failure to establish intravenous/intraosseous access	2 (2%)	2 (3%)	0 (0%)
Difficulties to move patient from scene of arrest *	2 (2%)	2 (3%)	0 (0%)

In-hospital (patients with cannulation initiated)	***N* = 52**^a^	***N* = 34**	***N* = 18**^a^
Difficult access for cannulation	6 (12%)	5 (15%)	1 (5%)
Severe bleeding at access site requiring intervention^a^	2 (4%)	1 (3%)	1 (5%)
Wrong vessel cannulated	0 (0%)	0 (0%)	0 (0%)
Perforation of vessel	1 (2%)	0 (0%)	1 (5%)
Dissection of vessel	0 (0%)	0 (0%)	0 (0%)
Peripheral ischemia	0 (0%)	0 (0%)	0 (0%)
Insufficient ECMO-flow at ECMO initiation	2 (4%)	2 (6%)	0 (0%)
Air embolism	1(2%)	1 (3%)	0(0%)

Patients admitted at ICU on ECMO	***N* = 23**	***N* = 19**	***N* = 4**
Severe bleeding requiring transfusion^b^	4 (17%)	2 (11%)	2 (50%)
Pulmonary oedema	2 (9%)	2 (11%)	0 (0%)
Renal failure requiring dialysis	6 (26%)	5 (26%)	1 (25%)
Sepsis	1 (4%)	1 (5%)	0 (0%)
Re-arrest	0 (0%)	0 (0%)	0 (0%)

a Three patients with missing values.

b Detailed description of the individual cases can be found in supplementary material.

Overall survival to hospital discharge for patients included in the study, achieving ROSC with and without ECPR, was 25% (*N* = 24/95).

## Discussion

### Main findings

The main finding of this prospective observational study is that the implementation of the ECPR protocol in the greater Stockholm area was feasible without any major, unexpected safety aspects, but did not meet the intended target time interval of 60 min from cardiac arrest to ECMO-initiation. The survival rates were consistent with previous reports from randomized trials although the majority of ECPR-survivors had a time from OHCA to ECMO-initiation of more than 60 min. The majority of survivors had a favourable neurological outcome. Rooms for improvement were cannulation times and patient selection.

### Feasibility, safety and time intervals

The proportion of correctly identified patients according to prespecified ECPR-criteria was high in our study and few major adverse events were reported. We aimed at achieving ECMO-initiation within 60 min from cardiac arrest considering dismal survival above this time limit.^15^, ^16^, ^17^ However, in our study only in 9% of cases had ECMO initiated within 60 min from OHCA. Half of the patients, 49%, arrived at the hospital within the time limit of 45 min from collapse but the time from hospital arrival to ECMO-initiation was far longer than the 15 min first aimed. Similar in-hospital difficulties were found in the recent INCEPTION-trial by Suverein et al.^10^ while the two other larger RCTs in the field of ECPR have demonstrated very fast cannulation times. While prehospital times are similar across the three RCTs, the in-hospital times differ. It thus seems that prehospital implementation is easier achieved compared to in-hospital as this includes more new challenging elements. As described in detail in the result section, other major safety issues were fewer than those reported from other ECPR-studies.^8^, ^10^

### ROSC before ECPR

We found that 22 patients (23%) attained ROSC before and without ECMO-initiation and a high proportion of these survived. This is consistent with current published RCTs^9^, ^10^ and important as it has been suggested that intra-arrest transportation reduces survival^18^. Even if ROSC-rates are much lower than in a more general OHCA population^19^ this is a very selected OHCA population with refractory arrest and an important finding. As we strive to improve the care for patients with refractory arrest only responding to ECPR it is important to not decrease the number of patients that would have attained ROSC if we had stayed on site continuing ACLS. It is encouraging that the proportion of patients achieving ROSC is comparable to the RCTs indicating its safety with transfer to hospital with ongoing CPR.

### Survival

The overall survival of 25% is in line with previous randomized trials. We present a higher survival rate among patients admitted on ECMO compared to the study by Suverein et. al. although the time durations are comparable. In our study 39% of patients admitted to ICU on ECMO survived (the majority with good neurological outcome with CPC 1–2) compared to 11% in the study by Suverein et al. Differences in patient selection and quality of care as well as involvement of one versus several centres might perhaps explain these differences.

The 60-minute time frame has been argued to be important for survival rates with ECPR.^15^, ^16^, ^17^ It is somewhat surprising that although ECMO-initiation in the vast majority of cases was later than 60 min following the cardiac arrest, survival rates are almost in line with the faster studies presented by Yannopoulos et al. and Belohlavek et al. It might be evident that the time frame is just one factor among other in the patient selection and that patient selection continues to be challenging. To put the number of participants, survivors, and safety issues in perspective, one could also in detail compare the results of the trial with a national reference group or other comparable matched local OHCA populations before ECPR-implementation. This was unfortunately not feasible at the moment of the current study. However, before launch of this study we performed a limited non‐published prestudy that identified that around 50 young patients (≤65 years) with witnessed VF OHCA died yearly despite ACLS in the greater Stockholm area, indicating the need to try to improve care for this selected population.

### Selection criteria

Choosing selection criteria for ECPR is challenging. In an ideal situation, the ECPR chain is activated only for patients finally found eligible for ECPR as excessive activation consumes valuable resources unnecessarily and reduces the cost-effectiveness. However, making the criteria too narrow excludes patients possible to save.^20^ We chose to include patients with PEA as initial rhythm because PEA has increased among patients with cardiac disease^21^ and because myocardial infarction and pulmonary embolism are common in these patients.^22^, ^23^ In our study, two of the survivors had pulmonary embolism as cause. However, patients with PEA have shown lower survival also in the context of ECPR^24^ which we also found. Furthermore, patients with PEA in our study had more comorbidities, were more rarely treated with PCI and included patients with aortic dissection (contraindicated in ECPR).

### Organisational challenges

ECPR implementation for prehospital OHCA is logistically demanding and it requires a multidisciplinary involvement at several levels of the health care system.^17^, ^24^ In our case we engaged a new team composition which required time and training to merge. In addition, one success of the program came from the early involvement of a large number of stakeholders and organizations with several years of discussions and preparations at prehospital, hospital and county level.

We implemented our protocol one month before the first case of COVID-19 was found in Sweden. The COVID-19 pandemic influenced our ability to implement in several ways including the ability to train and stimulate, high hospital workload, exclusion of ventilation in CPR and protective gear, which reduced tempo in reaching patients, as well as reduced focus on cardiac arrest in general.

### Limitations

Our study has several limitations. First, we do not present a control group making comparisons of outcomes difficult. Second, the narrow time criteria limited patients possible for inclusion which can introduce bias as patients with faster access to the hospital may differ from those with less access. Third, the number of patients included in the trial is limited, thus making conclusions and associations about time intervals and outcomes difficult. Fourth, the implementation of an ECPR program and the learning curve for those involved takes time, introducing a potential improved benefit over time; however, this was not evaluated in the current trial due to the limited time period. Fifth, even if we found few important major safety issues we cannot by certainty conclude that ECPR is of no harm to the patients.

## Conclusions

The implementation of an ECPR protocol in the greater Stockholm area was safe and feasible but did not meet the intended target time intervals from cardiac arrest to ECMO-initiation. Despite this, survival rates were similar to previous reports from randomised studies and the vast majority of survivors had more than 60 min from cardiac arrest to ECMO-initiation. Future work should focus on reducing cannulation times as well as improving selection criteria.

## Funding

The study was funded by the 10.13039/501100003793Swedish Heart Lung Foundation. We also received funding from Jolife AB/Stryker. Jolife AB/Stryker were not involved in any parts of the study, thus had no impact on study design, management, results nor manuscript.

## CRediT authorship contribution statement

**Lis Frykler Abazi:** Writing – review & editing, Writing – original draft, Visualization, Project administration, Methodology, Investigation, Formal analysis, Data curation. **Andreas Liliequist:** Writing – review & editing, Resources, Project administration, Methodology, Investigation. **Felix Böhm:** Writing – review & editing, Project administration, Methodology, Investigation, Conceptualization. **Magnus Hedberg:** Writing – review & editing, Project administration, Methodology, Investigation, Conceptualization. **Moa Simonsson:** Writing – review & editing, Project administration, Methodology, Investigation, Conceptualization. **Anders Bäckman:** Writing – review & editing, Project administration, Methodology, Investigation, Data curation, Conceptualization. **Malin Ax:** Writing – review & editing, Project administration, Methodology, Investigation, Conceptualization. **Frieder Braunschweig:** Writing – review & editing, Resources, Project administration, Methodology, Investigation, Conceptualization. **Linda Mellbin:** Writing – review & editing, Project administration, Methodology, Investigation, Conceptualization. **Rickard Linder:** Writing – review & editing, Methodology, Investigation. **Leif Svensson:** Writing – review & editing, Methodology, Investigation, Conceptualization. **Juliane Jurga:** Writing – review & editing, Project administration, Methodology, Investigation. **Per Nordberg:** Writing – review & editing, Writing – original draft, Project administration, Methodology, Investigation, Formal analysis, Conceptualization. **Mattias Ringh:** Writing – review & editing, Writing – original draft, Methodology, Investigation, Formal analysis, Conceptualization. **Sune Forsberg:** Writing – review & editing, Writing – original draft, Resources, Project administration, Methodology, Investigation, Formal analysis, Conceptualization. **Jacob Hollenberg:** Writing – review & editing, Writing – original draft, Resources, Project administration, Methodology, Investigation, Formal analysis, Conceptualization.

## Declaration of competing interest

The authors declare the following financial interests/personal relationships which may be considered as potential competing interests: [Jolife AB/Stryker sponsored the study with the loan of LUCAS3 and research funding. Jolife AB/Stryker were not involved in any parts of the study, thus had no impact on study design, management, results nor manuscript.].
