# Supplementary material for: Implementation of an extracorporeal resuscitation (ECPR) program for out-of-hospital cardiac arrest in Stockholm, Sweden: Feasibility, safety, and outcome
Source: Resusc Plus. 2024 Mar 7;18:100596. doi: 10.1016/j.resplu.2024.100596 (PMC10937228; doi:10.1016/j.resplu.2024.100596)
Supplement: Supplementary data 1 [file mmc1.pdf]

Implementation of an extracorporeal resuscitation (ECPR) program for out-of-hospital cardiac arrest in Stockholm, Sweden: feasibility, safety, and outcome

Supplemental digital content

Table of contents

eTable 1. Criteria for activating the ECPR team and contraindications to ECMO-initiation 3

eTable 2. Outcomes 3

eTable 3. Details on reported adverse events. 4

eFigure 1. Inclusion of patients by month and year 5

eFigure 2. Flowchart of patients with reported reasons for exclusion or no initiation of cannulation 6

Appendix 1. More detailed description of methods 7

Appendix 2. Appendix 2. Prehospital and hospital case report forms. 10

eTable 1. Criteria for activating the ECPR team and contraindications to ECMO-initiation

Criteria for activating ECPR team
Witnessed OHCA
Bystander CPR within 5 min
Initial rhythm VF/pVT or PEA
Age 18-65 years
Absence of terminal malignancy, neurologic disability, cardiac or pulmonary disease

Contraindication for ECMO-initiation
Unwitnessed arrest
Asystole as initial rhythm
Return of spontaneous circulation
Lactate >15 at arrival on the catheterization lab
$EtCO_2$ <1,3
Uncontrolled bleeding
Free aortic insufficiency or aortic dissection
ECMO initiation >60 min from arrest

OHCA = out-of-hospital cardiac arrest. CPR = cardiopulmonary resuscitation. VF = ventricular fibrillation. pVT = pulseless ventricular tachycardia. PEA = pulseless electrical activity. $EtCO_2$ = End-tidal carbon dioxide.

eTable 2. Outcomes

Primary outcomes – Safety and feasibility	Secondary clinical outcomes
Proportion of patients arriving at hospital within 45 min	Timing of critical steps and key interventions such as angiography, PCI, target management
Proportion of patients with successful cannulation	Proportion of patients treated with the key interventions
Proportion of patients with ECMO-flow within 60 min	Overall survival
Proportion of patients achieving ROSC before ECMO	Overall survival with good neurological function (CPC 1-2 and mRS 0-2)
Safety deviations	
Prehospital and in-hospital logistic challenges	

PCI = Percutaneous coronary intervention
CPC = Cerebral performance category

eTable 3. Details on reported adverse events.

Patient complications potentially caused by CPR

1. One patient with haemorrhage in pleural space and liver laceration.
2. One patient with liver laceration only.
3. Three patients with pulmonary bleedings.

Difficulties to move patient from scene of arrest

1. A challenging scene of patient extraction from the place of cardiac arrest was described. The extraction included moving the patient through a small and unstable temporary staircase which prolonged the transportation time but also put the patient and staff at risk.
2. One patient transportation was prolonged because the patient had to be carried a long distance by foot and stretcher between the site of cardiac arrest and the helicopter landing site.

Severe bleeding requiring* transfusion**

1. One patient with liver lacerations caused by the mechanical compressions
2. One patient with pulmonary bleeding
3. One patient with bleeding in the bowel secondary to vascular perforation.
4. One patient with bleeding in the groin that required surgical intervention.

Severe bleeding at access site requiring intervention****

1. One distal catheter dislocating causing a hematoma in the groin as well as a vascular aneurysm in the same vessel after decannulation.
2. One patient with bleeding in the groin that required surgical intervention.

eFigure 1. Inclusion of patients by month and year

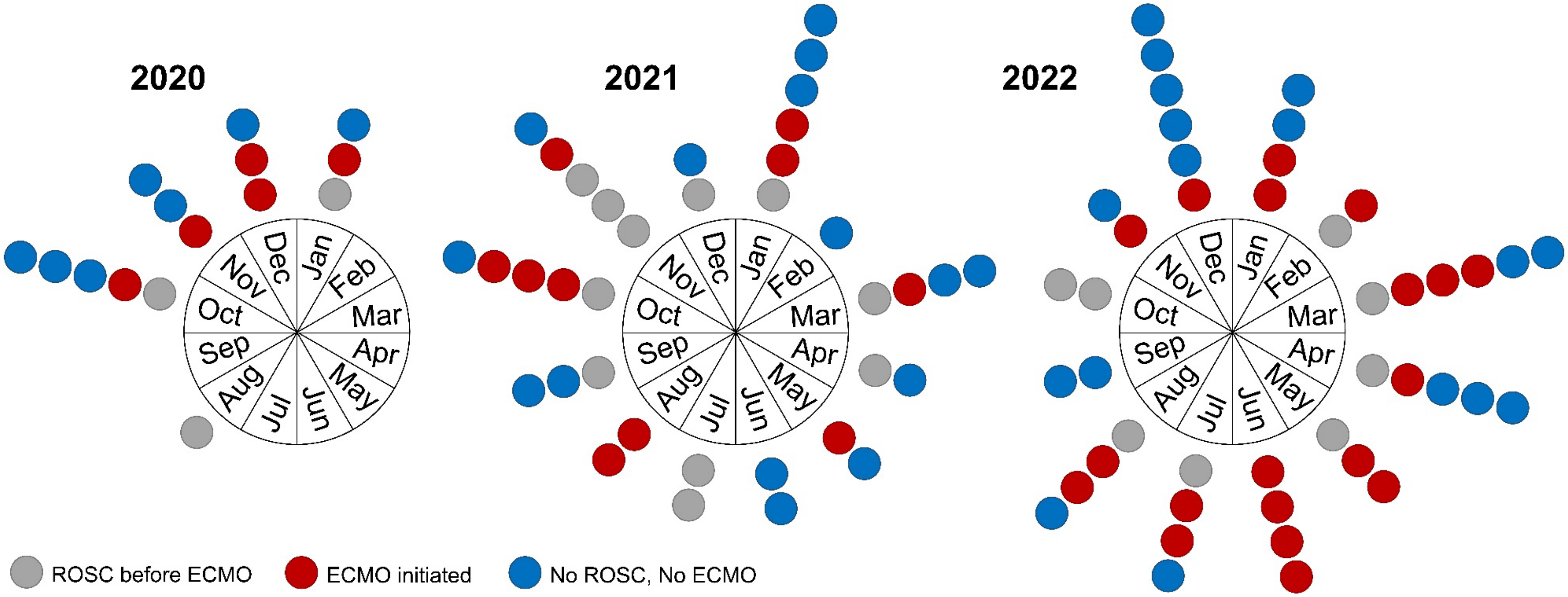

eFigure 2. Flowchart of patients with reported reasons for exclusion or no initiation of cannulation. Of 120 patients, 73 were considered eligible for ECMO. For details see Figure 1 in main manuscript.

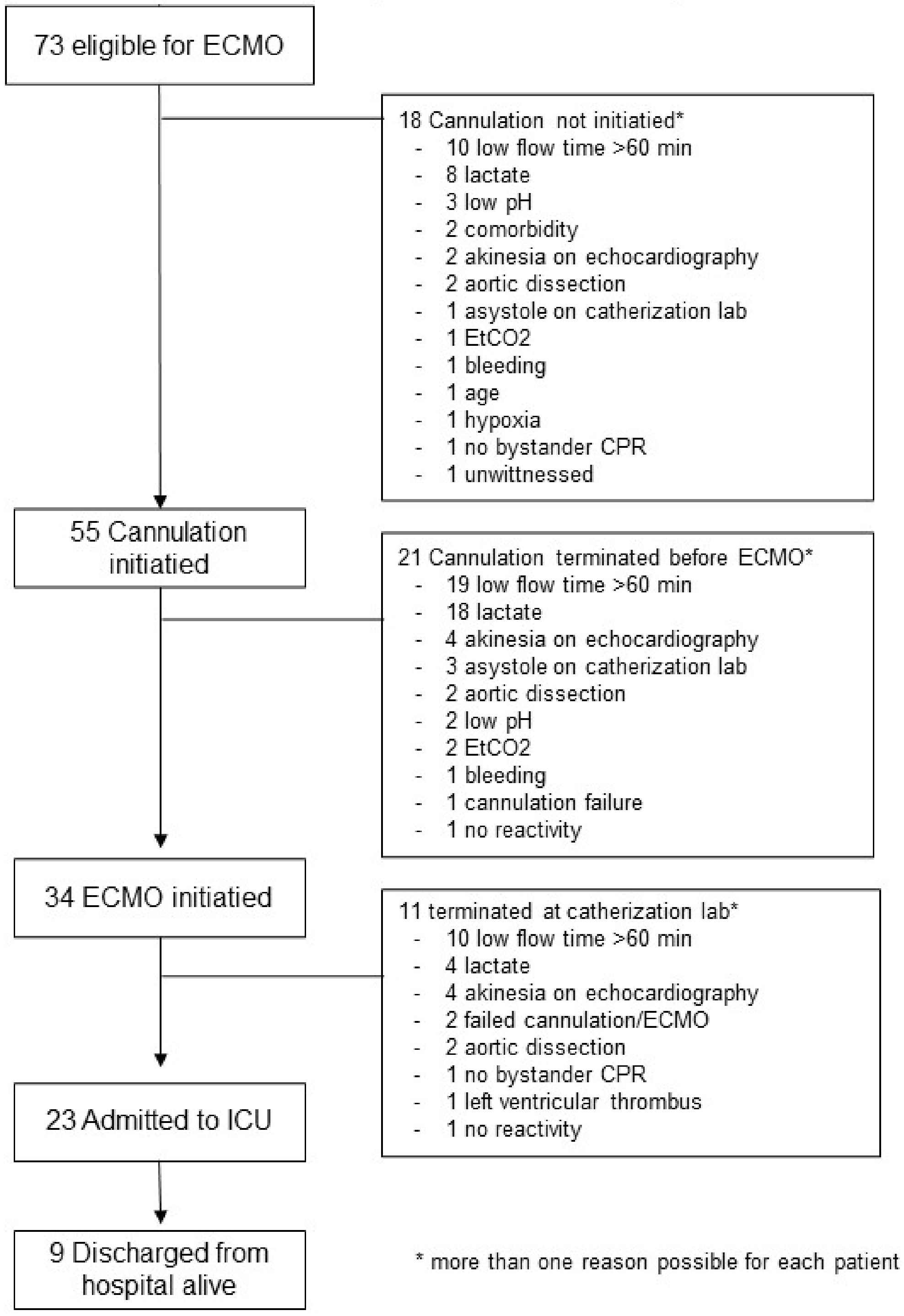

Appendix 1. More detailed description of methods

Setting

This observational study reports findings from the implementation of an extracorporeal cardiopulmonary resuscitation (ECPR) protocol in the greater Stockholm area, Sweden, with a population of 2.4 million (13) and an area of 6 514 km^2 (14). Six hospitals serve the greater Stockholm. Extracorporeal membrane oxygenation (ECMO) is available at one hospital, Karolinska University Hospital, in both the thoracic intensive care unit (ICU) and a dedicated ECMO center in the same hospital. The emergency medical system (EMS) share one common dispatch centre (SOS Alarm AB) and one common two-tier EMS. The first tier includes eighty-three ambulances capable of providing advanced cardiac life support (ACLS). The second tier consist of three physician-staffed rapid response vehicles and two helicopters capable of performing more advanced ACLS. Before the implementation of the ECPR protocol the physician-staffed units and the helicopters were equipped with the same mechanical compression device (LUCAS3^{TM}, Jolife AB/Stryker. Lund, Sweden). Access to physician-staffed units is limited at night because only one of the three physician-staffed unites operates at 21:00 to 07:00. All emergency healthcare is publicly financed in Sweden.

Treatment prior to ECPR protocol implementation

Prior to the start of the project, ECPR for out-of-hospital cardiac arrest (OHCA) was initiated on very rare occasions and patients with OHCA were primarily treated on site by ambulance personnel. In cases of suspected cardiac arrest two units were normally dispatched, one of which was a physician staffed unit or a helicopter unit, provided they were available. Transportation to hospital was assessed on individual basis at the discretion of the attending staff, for specific cases such as young patient, hypothermia, intoxication etc. and transportation made to the emergency department at the nearest hospital. Upon arrival at the emergency department a central ECMO team at Karolinska University hospital could be consulted for evaluation which commonly could not be initiated until after arrival at the emergency department. Treatment followed current guidelines on ACLS.

Establishment of an ECPR chain of care and treatment protocol

Prehospital care

The ECPR protocol modified the prehospital treatment logistics for the selected group of refractory cardiac arrest patients in several ways:

1) For patients who met the inclusion and exclusion criteria, the onsite time was intentionally reduced and focus was shifted to early transportation with ongoing CPR rather than staying on site. This meant that transportation to the hospital was no longer only at the discretion of the attending staff but prompted for all eligible cases. When all criteria were met, patients were transported as soon as possible.

2) The physician-staffed units and the helicopter unit were equipped with the same mechanical LUCAS chest compression device. This allowed for continuous CPR during transport. The second-tier staff were all trained on the use of mechanical compression device as well as treatment protocol details prior to the beginning of the ECPR protocol.

3) If all pre-hospital inclusion criteria were met the in-hospital newly established ECPR team was alerted by the ambulance physician-staffed unit or helicopter unit. The EMS crews were instructed to bypass the nearest hospital on their way to the ECPR site at Karolinska University Hospital and the patients were taken directly to the coronary catheterization lab rather than the emergency department. If return of spontaneous circulation (ROSC) was achieved during transportation and

ECPR no longer required, the EMS crew was instructed to redirect to the nearest hospital. Standard ACLS including defibrillation and drugs was continued according to guidelines.

In-hospital care

Veno-arterial (VA) ECMO is a well-established treatment at the Karolinska University Hospital in Stockholm. ECPR has previously been performed on in-hospital cardiac arrest and selected rare cases of OHCA but without a standardized protocol for prehospital identification of patients, management, and transport. In this study, the patients were taken directly to the coronary catheterization lab where the in-hospital ECPR team awaited. The team included a non-invasive cardiologist, a percutaneous coronary intervention (PCI) operator, a ECMO-experienced intensivist, a perfusionist as well as a cardiac catheterization lab nurse, a thorax surgery nurse, a nurse anaesthetist and assistant nurses.

In general, a short echocardiography (transoesophageal and/or transthoracic) was performed to rule out severe aortic regurgitation, cardiac tamponade and aortic dissection, and the medical chart reviewed for terminal illnesses. There were no additional absolute criteria decided to abstain from ECMO.

Cannulation was performed by the attending PCI operator by percutaneous ultrasound-guided technique. After initiating VA-ECMO, coronary angiography was performed and the patient was treated with PCI in accordance with European Society of Cardiology (ESC) guidelines. This means that culprit lesion PCI was performed, and any non-culprit lesions were treated as a staged procedure, usually prior to weaning from ECMO. Following index coronary angiography/PCI, a distal perfusion catheter was put in place before the patient left the catheterization lab. The attending PCI operator and the perfusionist were only on site at the hospital during working hours. Outside of regular working hours they were summoned once the ECPR-protocol was activated with a response time of up to 30 min.

The implementation of the ECPR protocol included hands-on team training of the ECPR team at the hospital as well as simulations of the entire treatment chain from prehospital care to cannulation and ECMO-initiation. These trainings sessions and simulations were performed prior to start of the study. The PCI operators were encouraged to participate in elective cases of cannulation as well as simulations to learn the technique.

Prestudy and start of the project

First, a non-published prestudy was performed that identified that around 50 young patients (≤65 years) with witnessed VF OHCA died yearly despite ACLS in the greater Stockholm area. Second, a five year process followed in which a large number of stakeholders and organizations were included. These included the regional dispatch center, all four ambulance organizations, regional research organizations, 6 hospitals (with participants of cardiology, intensive care, emergency medicine, chief medical officers and all CEOs) as well as chief medical officers and decision makers at the County level. A great number of medical, logistical, economic and ethical issues were discussed. After a final agreement about inclusion and exclusion criteria was made and the ECPR organization was put into place in January 2020 which included a novel complete treatment chain prehospitally into the hospital level.

Data collection

Data collection in the present study derives from a study-specific prehospital and a hospital case report form (CRF), a pre-hospital EMS database and electronic medical records.

The prehospital CRF was completed by the second-tier unit at the time of the cardiac arrest. It collected key data on ECPR protocol criteria including inclusion and exclusion criteria, prehospital timing of the interventions and events, and prehospital medication. These interventions and events included time of cardiac arrest, activation of ECPR team, departure from scene, arrival at hospital, ROSC (when applicable), intubation, mechanical chest compression, number of shocks, medication doses etc. After arrival at the coronary catheterization lab the second study-specific hospital CRF was completed by a member of the ECPR team on call. The hospital CRF collected data on the timing of the interventions and events related to the coronary catheterization lab including start of cannulation, ECMO-initiation, coronary angiography, percutaneous coronary intervention, reasons for not cannulating, etc. Both the prehospital and the hospital CRFs collected data on protocol deviations and adverse events.

Data on comorbidities, aetiology, duration of ECMO and hospitalization, adverse events at ICU and outcome were collected using electronic medical records. In cases where an autopsy was performed by the National Board of Forensic Medicine and thereby not visible in the electronic medical records, the report was manually retrieved for aetiology. The final cause of cardiac arrest was collected from medical charts based on what the responsible clinicians stated as the cause in each case. Data on EMS arrival time was retrieved from the prehospital electronic medical records. When data in the CRFs was missing, the electronic medical records were screened for these details. Outcome was also evaluated using the electronic medical records and the best score of CPC within the first year was used. When best CPC was difficult to evaluate using the electronic medical records the patient was contacted by phone. The data collection involving electronic medical records was conducted by the first author. Patients with missing data points were only excluded for the relevant analyses.

Appendix 2. Prehospital and hospital case report forms.

PREHOSPITAL GUIDELINES FOR REFRACTORY CARDIAC ARREST /ECPR-ALARM

CRITERIA

Witnessed arrest	☐ Yes	☐ No
Bystander CPR initiated within 5 min	☐ Yes	☐ No
VT/VF ☐ or PEA ☐ as initial rythm	☐ Yes	☐ No
Age 18 – 65	☐ Yes	☐ No
No known advanced malignancy, sever neurology, cardiac or pulmonary disease or ongoing bleeding	☐ Yes	☐ No
Refractory cardiac arrest after 6 min of ACLS	☐ Yes	☐ No

YES on all criteria: activate ECPR-team via emergency department Karolinska. Call 08 -517 788 92 – state expected arrival time (If no answer, call 08 – 517 711 77 or through Rakel 339-1040)

IN DOUBTFULL CASES DISCUSS WITH TICU 08 - 517 784 14

TIME OF RREST TIME: ____________

DATE: ____________

SOS CASE NUMBER: ____________

PERSONAL NUMBER: ____________

NAME: ____________

SECOND TIER UNIT:
☐ ALB 339-9910 ☐ HKP 379-5980
☐ ALB 339-9930 ☐ HKP 379-5840
☐ ALB 339-9950

Adverse event: ☐ Yes ☐ No

DESCRIBE BRIEFLY

____

INTERVENTIONS ON SITE

Contact with Karolinska Time: ______

Contact with TICU: ☐ Yes ☐ No Time: ______

Start of LUCAS Time: ______

Intubation during CPR (maximum 1 attempt 30 s else LMA)

Time of intubation Time: ______

Fast departure! Time of departure Time: ______

INTERVENTIONS DURING TRANSPORTATION

Notify emergency department Karolinska ; "ECPR alarm"

No stop for iv-line insertion/medication/intubation etc

ACLS according to guidelines. Cut clothes.

ROSC during transport: ☐ Yes ☐ No
Time: ______

Meassure end tidal carbon dioxid (MINUTES. AFTER INTUBATION)

After LMA/ Intubation	Start of LUCAS	5 min	10 min	15 min	20 min	At termination	At ROSC	At Hospital arrival
--------kPa	--------kPa	--------kPa	--------kPa	--------kPa	--------kPa	--------kPa	--------kPa	--------kPa

ARRIVAL AT HOSPITAL

Time arrival hospital: Time: ________

Status at arrival: ☐ VT/VF ☐ PEA ☐ Asystole ☐ ROSC

PREHOSPITAL

Number of defibrillations: ________

Total dose adrenaline: ____________mg

Total dose amiodarone: ____________mg

REFRACTORY CARDIAC ARREST/ECPR-ALARM
- HOSPITAL CARE

CRITERIA PREHOSPITAL UNIT

Witnessed arrest	☐ Yes	☐ No
Bystander CPR initiated within 5 min	☐ Yes	☐ No
VT/VF ☐ or PEA ☐ as initial rythm	☐ Yes	☐ No
Age 18 – 65	☐ Yes	☐ No
No known advanced malignancy, sever neurology, cardiac or pulmonary disease or ongoing bleeding	☐ Yes	☐ No
Refractory cardiac arrest after 6 min of ACLS	☐ Yes	☐ No

PERSONAL NUMBER: ____________

NAME: ____________

DATE: ____________

TIME OF CARDIAC ARREST
Time: ____________

AT HOSPITAL

Arrival catheterization lab Time: ______

Lactate =________ (< 15)	☐ Yes	☐ No
No ROSC	☐ Yes	☐ No

If YES on all criteria
FAST CANNULATION AND INITIATE ECMO WITHIN 60 MIN FROM ARREST

OTHER RELEVANT INFORMATION
____
____
____
____
____

CANNULATION

Decission to cannulate: ☐ Yes ☐ No

Cannulatiom initiated Time:____ ECMO initiated Time:____

EKO performed, presens of tamponade excluded: ☐ Yes ☐ No

ADVERSE EVENTS

Bleeding	☐ Yes ☐ No	Wrong vessel	☐ Yes ☐ No
Perforation	☐ Yes ☐ No	Peripheral ischemia	☐ Yes ☐ No
Difficult access	☐ Yes ☐ No	Arterial dissection	☐ Yes ☐ No

Return of circulation (>10 minutes without CPR) Time: ____

IF DECISSION TO **NOT** CANNULATE/INITIATE ECMO. SPECIFY REASON BELOW.
____
____
____
____
____

ANGIOGRAPHY

Decision to perform angiography? ☐ Yes ☐ No

Angiography initiated Time:____ terminated Time:____

PCI performed: ☐ Yes ☐ No

AT ICU

Patient admitted alive to ICU ☐ Yes ☐ No

Arrival at ICU Time:____

TTM initiated Kl:______ Target temperature (32-36°C): ____°C

ECMO terminate Datum:________ Kl:______

Left ventricular function meassured with echo at ICU arrival: estimated EF: ______ %

at 72 hours (after ICU arrival): estimated EF: ______ %
